# A New Fungal Endophyte, *Scolecobasidium humicola*, Promotes Tomato Growth under Organic Nitrogen Conditions

**DOI:** 10.1371/journal.pone.0078746

**Published:** 2013-11-01

**Authors:** Rola S. Mahmoud, Kazuhiko Narisawa

**Affiliations:** 1 United Graduate School of Agricultural Science, Tokyo University of Agriculture and Technology, Fuchu-shi, Tokyo, Japan; 2 College of Agriculture, Ibaraki University, Ami machi, Ibaraki, Japan; Volcani Center, Israel

## Abstract

A new fungal endophyte, *Scolecobasidium humicola*, was identified as a common dark septate endophytic fungal (DSE) species under both natural and agricultural conditions. This fungus was found to grow endophylically in the roots of tomato seedlings. Light microscopy of cross-sections of colonized tomato roots showed that the intercellular, pigmented hyphae of the fungus were mostly limited to the epidermal layer and formed outer mantle-like structures. Two isolates of *S. humicola*, H2-2 and F1-3, have shown the ability to increase plant biomass with an organic nitrogen source. This finding is the first report of *S. humicola* as an endophyte and could help to improve plant growth with organic nitrogen sources.

## Introduction

In the last few decades, interest in organic farming has increased all over the world; however, only 0.9% of the world’s agricultural lands are organic [1]. Organic farming practices aim to establish stable production systems with concern for nature, in which the use of chemical nitrogen fertilizers, synthetic pesticides and growth-promoting chemicals is not allowed [2]. The input of nutrients in organic fields generally relies on organic fertilizers, manure, green manure, and/or crop residues; however, the balance of N mineralization/immobilization processes depends on the nature of these substrates and on the ecological conditions of each agro-system, which limit plant uptake and growth, especially at times of peak crop demand [3]. Recently, an interesting finding about plant nitrogen utilization in the forest ecosystem was reported [4]. It was shown that the largest pool of N is typically organic, e.g., amino acids and proteins are among the most abundant forms of organic N in the soil, comprising 80% of the soil N supply, while ammonium and nitrate contribute only 10%. This finding undoubtedly showed that symbiotic fungi, such as mycorhizal fungi, which can enhance host plant growth by improving N nutrition, are extremely significant in the forest ecosystem. This can be achieved via an increase in the absorptive surface area provided by fungal hyphae, greater uptake efficiency or by increasing access to various N sources that are unavailable to non-mycorrhizal plants [5,6]. However the ability of mycorrhizal fungi to maintain these benefits for their host plant under some field conditions common in industrialized agriculture is considered to be limited [7].

Dark septate endophytic fungi (DSE), which are a miscellaneous group of ascomycetes colonize root tissues intracellularly and intercellularly without causing apparent negative effects on the host plant [8–10]. DSE associations have been recognized in approx. 600 plant species of 320 genera in 114 families, including non-mycorrhizal plant species [11]. DSE may benefit their host plants by facilitating the uptake of plant mineral nutrients, including P, N and water [12–15], and suppressing infection by plant pathogens [16–18]. Many aspects of their ecological roles remain unclear although several studies had focused on the abundance of DSE in different habitats. For that reason, the most suitable utilization method of DSE for organic farming is still unclear.

In our study, we hypothesized that natural and agriculture systems might share similar DSE species, and DSE could play a role in supporting plant growth under these agriculture systems, especially the organic farming system. In order to prove this hypothesis, key DSE species were isolated and identified in both natural and agriculture ecosystems. Here, we describe the features or identity of the selected key DSE species that were effective in supporting tomato growth with different organic nitrogen sources.

## Materials and Methods

### Sample collection and fungal isolation

Soil samples were collected in October 2010 from a forest, organic field and conventional field at the Field Science Center of College of Agriculture, Ibaraki University Japan. Three soil samples (approximately 300 ml) were collected at 0-20 cm depth from each site and were kept in polyethylene bags and stored at 4°C for a maximum of one month prior to utilization.

Composite soil was prepared to bait endophytic fungi as described by Narisawa et al. [17]. Each soil sample was combined and mixed with autoclaved potting soil (Peat pot; Kureha Chemical Industry Co., Tokyo, Japan) at the ratio of 1:2 (v/v). The seeds of tomato *Solanum lycopersicum* cv. Gohobi (Sakata Seed, Yokohama, Japan) and Chinese cabbage *Brassica campestris* cv. Musou (Takii Seed, Kyoto, Japan) were surface sterilized by immersion in a 70% solution of ethanol for 30 seconds, followed by a solution of sodium hypochlorite (2% available chlorine) for 1.5 minutes. Then seeds were rinsed three times with sterilized distillated water, dried overnight and placed on 1.5% water agar medium (15 g Bacto agar [Difco Laboratories, Detroit, MI] for 1 liter distilled water) in Petri dishes. After 4 days, the axenically grown seedlings were transplanted (three seedlings per pot) in to 90-mm diameter pots containing 100 ml composite soil. Each collection site was considered as a bloc containing three replicates for each soil sample and plant species. Seedlings were grown under greenhouse conditions with the temperature reaching 25°C. After three months, the roots collected from young tomato and Chinese cabbage plants in each replicate were washed with running tap water to remove debris and cut into approximately 1-cm segments. Fifteen root segments were chosen in random from each bait plant, washed three times in a 0.005% solution of Tween 20 (J.T. Baker Chemical Co., Philipsburg, NJ), and rinsed three times with sterile distilled water (SDW). The segments were air-dried overnight and plated on nutrient agar containing 25 g. L^-1^ corn meal (infusion form; Difco Laboratories) and 15 g. L^-1^ Bacto agar (Difco Laboratories). These plated roots were incubated for 3 weeks at room temperature (approximately 23°C).

### Morphological observation and identification

Fungal isolates were identified on the basis of microscopic morphology. The pure fungal culture was grown at room temperature on 55-mm diameter Petri dishes containing half-strength cornmeal malt yeast extract agar (1/2 CMMY, 25 g corn meal (infusion from Difco), 15 g Bacto agar (Difco), 10g malt extract (Difco), 2g yeast extract (Difco), for 1 L distilled water). To provide good observation conditions, slide cultures were made. Small pieces, approximately 3 x 3 mm, of Publum agar [Mead Johnson mixed Publum; Canadian Post Corporation, Ontario, Canada, 25 g; Bacto agar, 5 g; MilliQ water, 250 ml] were sandwiched between two 18 x 18-mm cover glasses (Matsunami Class Ind., Osaka, Japan) and placed in a 9-cm water agar plate to provide humidity. After 2-4 weeks, when the culture had grown sufficiently, the Publum agar was carefully removed and cover glasses were mounted on 76 x 26 mm micro slide glasses using PVLG (Polyvinyl alcohol, 16.6 g; Lactic acid (Wako Chemical Ind., Osaka, Japan), 100 ml; glycerin (Wako Chemical Ind., Osaka, Japan), 10 ml; MilliQ water, 100 ml) mounting medium. Conidiogenous cells and conidia were measured under a light microscope (BX51; Olympus, Tokyo, Japan) with UPlanFLN FLN100x/1.30 Oil.

### DNA extraction, amplification, sequencing and analysis

Fungal DNA were extracted using a PrepMan TM Ultra Extraction Kit (Applied Biosystem, Foster City, CA, USA) according to the manufacturer’s protocol, The fungal isolate was identified by sequencing the internal transcribed region (ITS) of the 18S rDNA, using universal primers ITS-1 (5'-TCC GTA GGT GAA CCT GCG G-3') and ITS-4 (5'-TCC TCC GCT TAT TGA TAT GC-3'). Fifty microliters of PCR mixture contained 0.2 μM concentration of each primer, 0.2 mM of each deoxynucleoside triphosphate, 10× Ex Taq buffer (TaKaRa Bio, Otsu, Japan) and 0.25 U of Ex Taq DNA polymerase (TaKaRa Bio), and 50ng DNA template. The reaction cycle consisted of initial denaturation at 94 °C for 4 min followed by 35 cycles of denaturation at 94 °C for 35 sec, annealing at 52 °C for 55 sec and extension at 72 °C for 2 min, and final extension at 72 °C for 10 min. The PCR products were sequenced using a model 3130x DNA sequencer (Applied Biosystems, Foster City, CA, USA) and the ABI PRISM TM Big Dye Terminator v3.1 Cycle Sequencing Ready Reaction Kit (Applied Biosystems). The primers used for sequence determination were ITS1F and ITS4R. The determined sequences were analyzed using MEGA version 5.05 and compared with similar DNA sequences retrieved from the DDBJ/EMBL/GenBank databases using the NCBIBLAST program. 

### Pathogenicity screening

In order to distinguish non-pathogenic fungi from pathogenic and other saprotrophic fungi, 15 fungal isolates showing diverse morphology were grown on oat meal agar medium (OMA; 10 g oatmeal and 18 g Bacto agar) enriched with nutrients (1 g MgSO_4_·7H_2_O, 1.5 g KH_2_PO_4_, and 1 g NaNO_3_) per liter in Petri dishes (55-mm diameter). They were incubated at room temperature (approximately 23°C). After two weeks, 2-day-old Chinese cabbage seedlings (three seedlings per plate) were transplanted onto each fungal colony. The seedlings transplanted onto non-inoculated medium were used as a control. The whole set was placed into sterile culture pots (CB-1; As One, Osaka, Japan) and incubated in a growth chamber at 23°C under a 16-h photoperiod (180 mol m^-2^ s^-1^) for 2 weeks. Symptoms were evaluated according to an index of 0 to 3 (0: no visible symptoms; 1: light yellowing; 2: yellowing and late growth; 3: wilting or death). Plants were harvested and oven-dried at 60°C for 48 h and their dry weight was measured for comparison with control plants. 

### Endophyte screening

After successful pathogenicity testing, the efficacy of selected isolates to promote tomato growth was observed. The fungal isolates were grown on Petri dishes filled with oat meal agar medium supplemented with nutrients (MgSO_4_.7H_2_O, 1 g, KH_2_PO_4_ 1.5 g; and NaNO_3_ 1 g L^-1^). Surface sterilization of tomato seeds was performed as described for the pathogenicity testing. Inoculation was preformed as described above. 

### Endophyte screening for organic nitrogen sources

In order to identify the effect of nitrogen on fungal infection, a nitrogen source test was conducted. The selected fungal isolates were grown for 2 weeks in 6-cm Petri dishes filled with oat meal agar medium as described above but NaNO_3_, 1g L^-1^ was replaced by one of the selected nitrogen sources of amino acids, such as L-Valin, L-Phenylalanine, and L-Leucine (Wako Industries, Ltd., Japan), at the concentration of 20 mg L^-1^. Tomato seedlings (three seedlings per plate) were transplanted and inoculation was preformed as described previously. 

To determine the endophytic nature of the fungal isolate, infected hyphae of the inoculated fungi in 3-week-old tomato seedling roots were observed after washing, cross sectioned, and stained in 50% acetic acid solution containing 0.005% cotton blue under an Olympus BX50 microscope with UplanFI20 and 40/0.30 objectives (Olympus).

### Data analysis

The mean dry biomass of each treatment was calculated and analyzed with one-way ANOVA. Differences among treatment means were detected with Tukey’s honestly significant difference test (Tukey HSD).

## Results and Discussion

### Fungal isolation

Two hundred and five fungal isolates were obtained from 270 root segments of tomato and Chinese cabbage grown as bait plants in a mixed soil. Fifty-six percent of fungal isolates were isolated from Chinese cabbage, and 44 % were isolated from tomato. The dominant isolated fungi are species of *Fusarium* (22%). They were mostly isolated within 4 days of placing root segments on the medium. Other taxa, including *Scolecobasidium humicola* (4%), *Leptodontidium orchidicola* (1%), and *Phialocephala fortinii* (1%) were isolated in much smaller numbers from both plants. They were mostly isolated after one to three weeks. *Scolecobasidium humicola* were found at all three sites but *L. orchidicola* and *P. fortinii* only in the forest. *Phialocephala fortinii* and *L.orchidicola* are DSE fungi distributed in a wide geographical area in many alpine and subalpine habitats. In addition, *P. fortinii* is considered to be the dominant root endophyte in forests [19]. The genus of *Scolecobasidium* was first described by Abbott in 1927 as two species, *S. terreum* and *S. constrictum*. The genus includes soil-borne and saprotrophytic species from plant litter [20–23]. Our study indicates that *S. humicola* is a dark septate endophyte in forests and agriculture fields, and is able to improve plant growth in comparison with the control. A new endophytic species, *S. humicola*, was described for the first time in this paper.

### Endophyte screening

**Figure 1:**
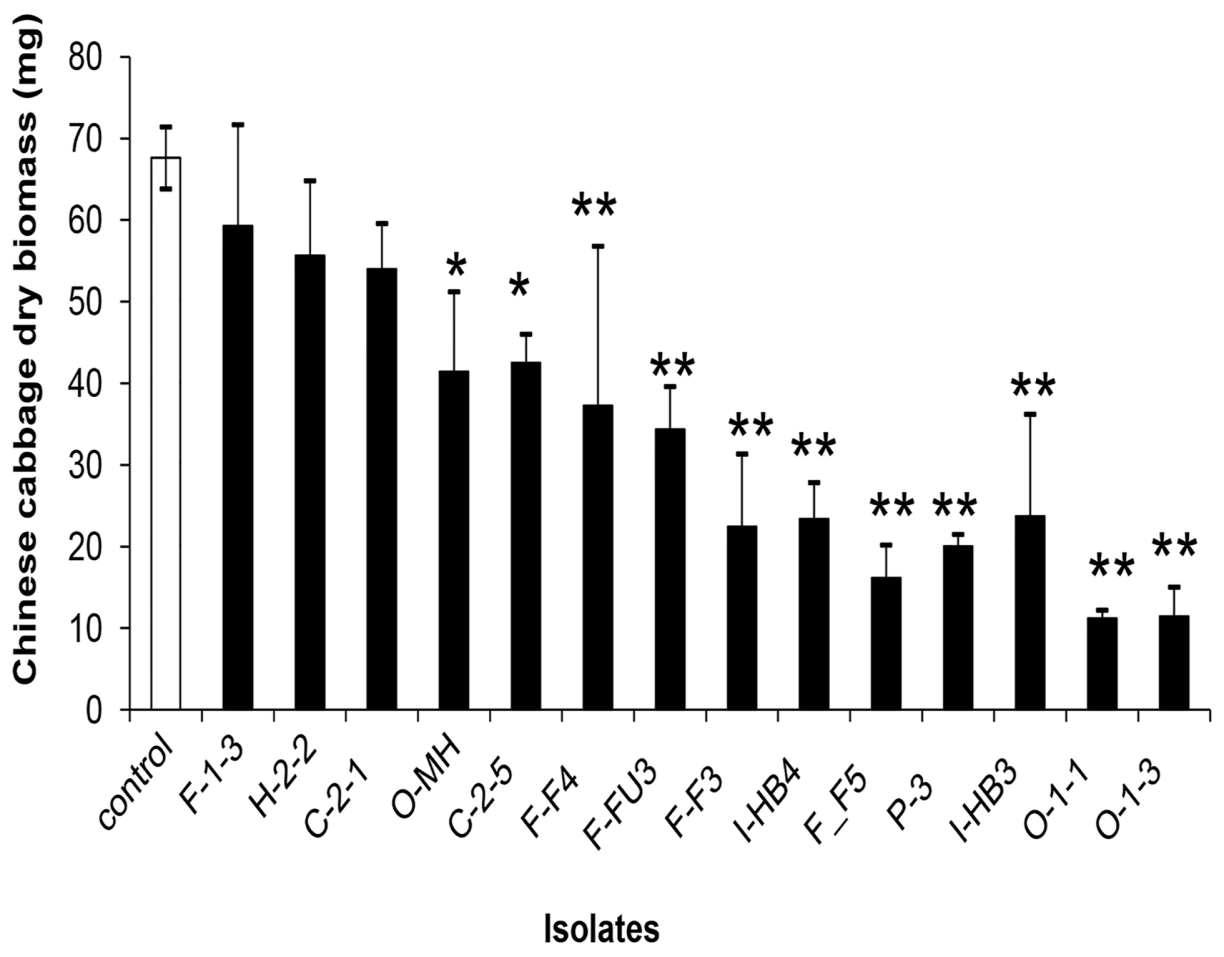
Dry weight of Chinese cabbage inoculated with different fungal isolates. Dry weight of Chinese cabbage seedlings grown on basal media (10 g oatmeal and 18 g Bacto agar, enriched with nutrients 1 g MgSO_4_·7H_2_O, 1.5 g KH_2_PO_4_, and 1 g NaNO_3_) inoculated with different fungal isolates. The filled columns represent the groups of selected fungi. Data are the means ± SE, n = 5. Asterisks represent significant differences between each treatment and the control (** P <0.01, * P <0.05) following Tukey’s honestly significant difference test.

To eliminate saprotrophic and/or pathogenic fungal isolates, Chinese cabbage seedlings were inoculated with 15 randomly selected isolates showing diverse morphology from each colonial morphology group. Seven isolates were originally obtained from the forest, 5 from a conventional field and 3 from an organic field. In the results of the inoculation test, only two of the 15 isolates tested (approximately 13 %) were not pathogenic to the Chinese cabbage seedlings. These two isolates, H2-2 and F1-3, caused no visible sign of disease or decay of the seedlings. The weight of dried plants was 56 ± 11 and 59 ± 14 mg for H2-2 and F1-3, respectively, and showed no significant difference compared to the control plants (68 ± 5 mg) (Figure 1). The most ineffective isolates (over 86%), once re-inoculated in to axenically-grown Chinese cabbage seedlings, caused extremely yellowing of leaves and suppression of plant growth (Figure 1). 

**Figure 2:**
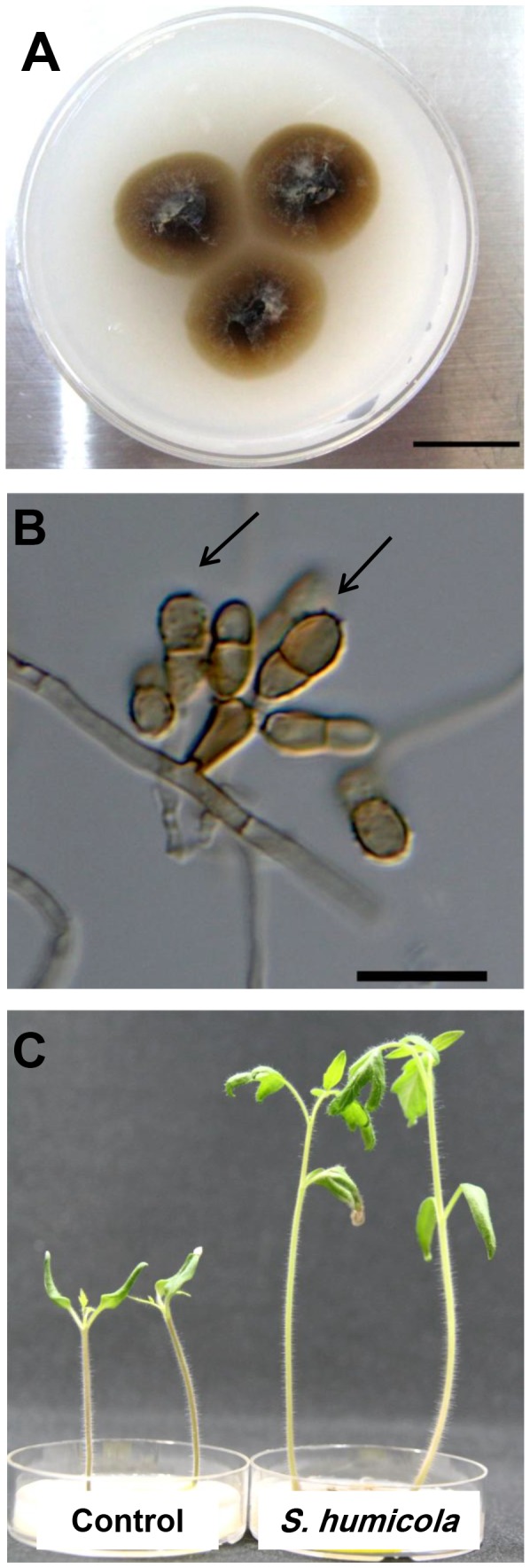
*Scolecobasidium humicola* colony and conidia. Colony (A) and a light micrograph (B) of *Scolecobasidium humicola* after 14 days at 23°C grown on OMA medium. Arrowhead indicates a micronematous conidiophore with rough-walled septate conidia (arrows). Bars: A = 14 mm, B = 20 µm. (C) Tomato seedlings grown on basal media (OMA) amended with L-Leucine amino acid, the control on the right , and inoculated tomato seedlings with *S.humicola* on the left.

Colonies of 3 isolates, H2-2, F1-3 and O-MH, on OMA medium were similarly dark brown. The colonial growth of the 3 isolates was similarly slow (up to 15 mm in diameter after 4-week incubation at 23 °C) (Figure 2a). The conidiophores were micronematous, and the conidia rough-walled with one to three septata (Figure 2b). Mecelial hyphae were hyaline to brown, septate-forming densely coiled hyphae, resembling microsclerotia. These morphological characteristics were identical among the 3 isolates and were in agreement with the genus of *Scolecobasidium*.

The ITS sequence of the three isolates showed 99%-100% similarity to *Scolecobasidium humicola* (NCBI/GenBank Accession No. DQ307332.1).

### Nitrogen source utilization and anatomic observations

**Figure 3:**
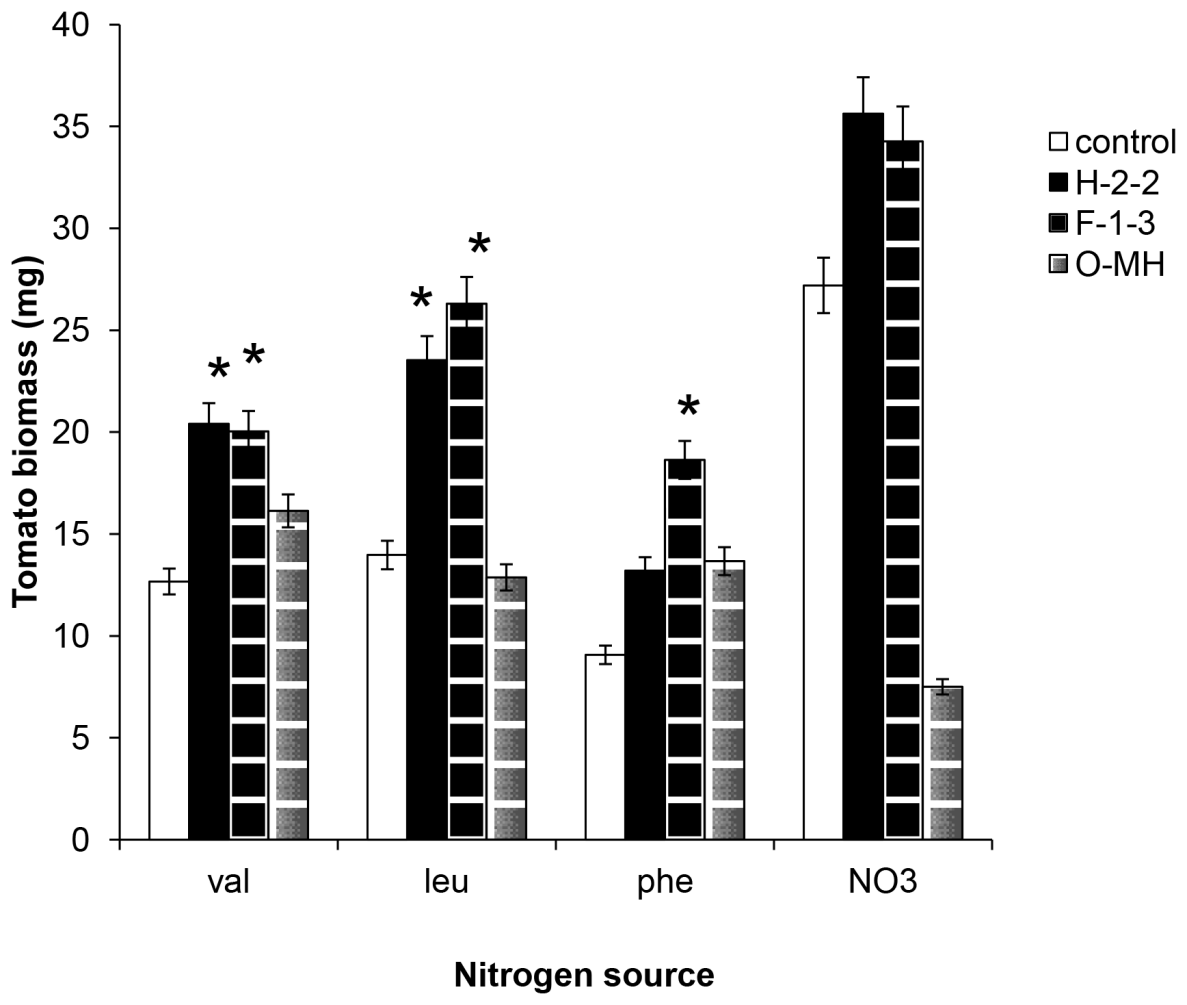
Dry weights of tomato inoculated with *Scolecobasidium humicola* . Dry weights of tomato seedlings grown on basal media (OMA) amended with four different nitrogen sources. white bar: control, black bar: *Scolecobasidium humicola* H2-2, black lines bar: *S. humicola* F1-3, and gray lines bar: *S. humicola* O-MH treatments. Data are the means ± SE, n = 5. Asterisks represent significant differences between each treatment and the control (P <0.05) following Tukey’s honestly significant difference test.

Uninoculated (control) plants could use NaNO_3_, but could not effectively use three amino acids (valin, leucine, and phenylalanine). Alternatively, plants treated with three isolates, H2-2, F1-3, and O-MH, of *S. humicola* were able to use all 3 amino acids (Figure 3). The treated plants with H2-2 and F1-3 isolates were able to grow well on the medium amended with NaNO_3_ as N source, but the treated plants with O-MH isolate showed yellowing leaves and decreased biomass by 50% on the same medium. The dry weight of treated plants with 3 isolates in Valin treatment was significantly high compared to the control. When phenylalanine was used with isolate F1-3, tomato plant biomass was significantly improved but not with 2 isolates H2-2 and O-MH. Optimum plant growth was observed when the plants were treated with H2-2 and F1-3 amended with leucine as a nitrogen source at 2% concentration.

Many studies have suggested the involvement of DSE fungi in nutrient uptake to host plants and improved plant growth under natural conditions, i.e. a forest [12,13,19]. In addition, DSE fungi have been isolated from agriculture fields under nutrient-stressed conditions [24,25]. We successfully showed that tomato plants treated with *S. humicola* H2-2 and F1-3 were able to use amino acids and the plant biomass increased in comparison with the control. This finding suggested that the ability of *S. humicola* as a plant growth-promoting fungus increased under organic nitrogen sources. This is not a unique feature of *S. humicola* as *Heteroconium chaetospira* could significantly improve plant biomass by transporting organic nitrogen to host plant [26]. Newsham [27] confirmed that the inoculated host plant with the selected DSE fungi responds more positively if there is no supply of available inorganic nitrogen.

**Figure 4:**
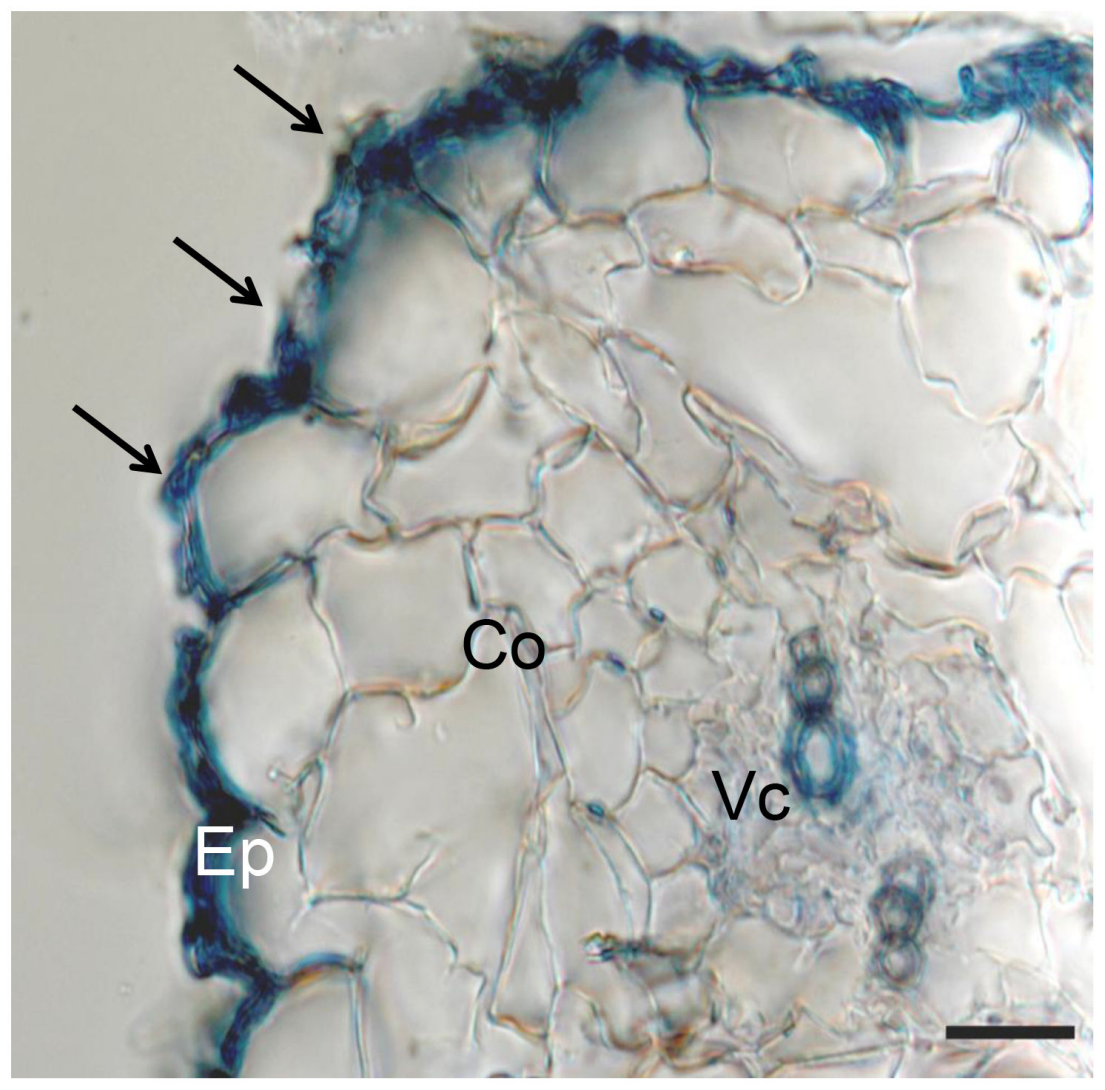
Interaction between tomato roots and *Scolecobasidium humicola*. Cross section of a tomato root stained with 0.005% cotton blue in 50% acetic acid 3 weeks after inoculation. The cortex (Co) mostly consists of three cell layers. Fungal hyphae can be seen on the root surface (arrows), within epidermal (Ep) cells. Vc = vascular cylinder. Bar = 20 µm.

To determine the endophytic nature of *S. humicola* isolate H2-2, anatomical observation of tomato roots under NaNO_3_ or leucine treatment was conducted. The hyphae of *S. humicola* colonized in epidermal cells heavily, but fungal hyphea were lightly colonized in outer cortical cells. No hyphae could be observed in the inner cortical cells or in the vascular cylinder under both treatments (Figure 4). The fungal hyphae only produced conidia and microsclerotia-like-structures on the root surface under NaNO_3_ treatment. 

In conclusion, although future detailed research is necessary to fully support our hypothesis, our study found for the first time that *S. humicola* is a common DSE species and may act as a key DSE species under both natural and agricultural conditions.
